# Pattern of uncontrolled allergic rhinitis in a hospital setting of Kinshasa, Democratic Republic of Congo

**DOI:** 10.1002/iid3.272

**Published:** 2019-09-18

**Authors:** Patricia K. Kakobo, Hilaire K. Kalala, Maguy M. Tshibola, Joseph K. Kelekele, Dieudonné T. Nyembue, Peter W. Hellings

**Affiliations:** ^1^ ENT Service Kinshasa University Hospital Kinshasa Democratic Republic of Congo; ^2^ Ophtalmology Service Kinshasa University Hospital Kinshasa Democratic Republic of Congo; ^3^ Department of Otorhinolaryngology and Head and Nose Surgery University Hospital of Leuven Leuven Belgium; ^4^ Department of Otorhinolaryngology, Academic Medical Center University of Amsterdam Amsterdam The Netherlands; ^5^ Upper Airways Research Laboratory University of Ghent Ghent Belgium

**Keywords:** allergens, clinical symptoms, Congo, Kinshasa, uncontrolled allergic rhinitis

## Abstract

**Aim:**

To determine the clinical and allergic features of uncontrolled allergic rhinitis (UCAR) in the Democratic Republic of Congo (DRC).

**Methods:**

Observational cross‐sectional study of 311 patients with UCAR. Allergic rhinitis was diagnosed clinically with sensitization to inhalant allergens and then confirmed by skin prick test. Severity was assessed using the Visual Analog Scale (VAS), with VAS scores greater than or equal to 5 used as cut off to determine uncontrolled status.

**Results:**

The mean age of UCAR patients was 30.7 ± 15.1 years and 66.9% of the patients were females. Three out of four patients had persistent UCAR while the remainder had intermittent symptoms. UCAR was associated with rhinosinusitis and asthma in 18.6% and 18% of the patients, respectively. Among UCAR patients, 95.2% were polysensitized. The allergens most frequently involved were mites (82%), cat (27.3%), and dog (26.7%). The most frequent symptoms were nasal congestion, sneezing, and runny nose. There were 44.4% of the patients treated with nasal corticosteroids and 33.1% with oral antihistamine (anti‐H1).

**Conclusions:**

This study reports on the clinical phenotype of UCAR in the DRC. The findings contribute to our understanding of UCAR in this population and may be used to implement strategies to reduce the prevalence and burden of UCAR in this setting.

## 1 INTRODUCTION

Allergic rhinitis (AR) is an inflammation of the nasal mucosa that is triggered by immunoglobulin E (IgE) in response to inhalation of allergens. It is characterized clinically by at least two nasal symptoms such as sneezing, rhinorrhea, and nasal congestion lasting at least 1 hour per day.^1^, ^2^

AR is a real problem of public health, given the high prevalence, the burden to patients and its major socioeconomic impact.^3^ AR affects up to 50% of the general population and its prevalence varies across regions.^4^, ^5^ However, reports show that African countries are more affected, with hospital‐based prevalence of 37.8% in Morocco,^6^ 33.0% in Zimbabwe,^7^ and 30.8% in the Democratic Republic of the Congo (DRC).^8^

Frequently, AR is unrecognized, under or wrongly diagnosed, and treated inadequately as a result of high frequency of self‐medication and management by non or less qualified personnel.^9^ While most patients are successfully controlled under treatment, approximately 20% of them will remain uncontrolled despite adequate treatment.^3^, ^10^ AR is uncontrolled when an adequately treated patient has a total score of 5 or greater using a Visual Analog Scale (VAS) for the combined naso‐ocular symptoms.^3^

Published data on uncontrolled allergic rhinitis (UCAR) remain scarce. In China, a prevalence of 27.7% of UCAR has been reported.^11^ A multicenter observational and prospective study conducted among 250 patients in Italy reported that more than 60% of them had an UCAR.^12^ Another multicenter observational study that enrolled 1482 patients across five European countries found 18.2% of UCAR cases regardless of the medications they were using.^13^ In a small sample of 88 patients recruited in rural and urban centers in Spain, symptoms of rhinitis and ocular symptoms were poorly controlled in 13.8%, 9.6%, and 8.0%, respectively.^14^ Despite the magnitude of AR in Africa, no data is available on UCAR on the continent.

The aim of this study was to describe the clinical pattern of patients diagnosed with UCAR and to identify the related allergens in a hospital setting in Kinshasa, DRC. Such information is needed for a better understanding of UCAR in this setting as it may help tailor strategies for diagnosis and management.

## 2 PATIENTS AND METHOD

### 2.1 Study population

This cross‐sectional prospective study was conducted between October 2018 and April 2019 in ear, nose, and throat (ENT) services of three hospitals of Kinshasa, including the University Hospital, Bondeko Village Center, and Monkole Hospital Center. Recruitment took place during regular outpatient visits. The study was approved by the School of Public Health Institutional Review Board (Approval #ESP/CE/082/2018). Oral informed consent was obtained from each patient before enrollment in the study and the study was conducted according to the Tenets of the Declaration of Helsinki. Participants were included in the study if they had (a) at least two visits to a physician for rhinitis during the last 5 years, (b) at least two AR symptoms including rhinorrhea, nasal congestion, and sneezing, and (c) a positive skin prick test (SPT) confirming the clinical suspicion of AR. Any patient with a total score of 5 or more on the VAS on the combined naso‐ocular symptoms after adequate treatment was defined as UCAR. They were excluded if at least one of the following criteria was met: age less than 10 years, having been seen at least two times in the past 5 years for the same condition, overall naso‐ocular VAS lower than 5 after treatment, negative SPT, disease duration less than 5 years, being immunocompromised, and refusal to provide informed consent. Adequate treatment was defined as the one based on Allergic Rhinitis and Its Impact on Asthma guideline.^1^ Any other treatment not following these guidelines was considered inadequate.

### 2.2 Questionnaire and clinical examination

The questionnaire included participants’ general information (sex, age, and duration of the rhinitis), disease‐related factors (environmental factors, cigarette smoke, and asthma), diagnosis‐related factors (sinusitis, nasal polyps, and incorrect diagnosis), treatment‐related factors (no compliance to treatment, treatment discontinuation without medical advice), and the investigator's assessment of the reasons for AR control failure. UCAR was classified as intermittent if symptoms lasted less than 4 days per week for less than 4 consecutive weeks per year. Patients with AR symptoms lasting more than 4 days per week for more than 4 consecutive weeks per year were diagnosed as having persistent UCAR.^15^

The VAS symptoms were evaluated using a ruler graded from 0 (no symptoms) to 10 (presence of worst symptoms).^16^ A total score of 5 or greater on all naso‐ocular symptoms was considered as indicative of UCAR.

### 2.3 Skin prick test

The SPT was performed using eight allergens (ALYOSTAL, Barcelona, Spain). The allergens tested included Dermatophagoides pteronyssinus, Dermatophagoides farinae, Blomia tropicalis, the 5‐grass pollen, dog's dander, cat's dander, *Alternaria alternata*, cockroach, peanut, soybean, and *Aspergillus* mix (*Aspergillus fumigatus*, *Aspergillus nidulans*, and *Aspergillus niger*). Histamine and physiologic serum were used as positive and negative controls, respectively. A drop of each allergen was placed on the forehand skin, then a sterile lancet needle was used to gently prick the skin for through of the allergen solution to enter below the surface of the skin. The response was evaluated 15 minutes later by measuring the size of the skin reaction. The test was considered positive greater than or equal to if the papule (raised bump) measured greater than or equal to 3 mm or greater in diameter or greater than or equal to the half of control the half that of positive control.^17^

### 2.4 Statistical analysis

Data were analyzed using Epidata version 3.1 (EpiData Association) and SPSS version 21 (IBM). Qualitative and qualitative variables were summarized as proportions and means ± standard deviation, respectively.

## 3 RESULTS

A total of 311 patients aged from 10 to 73 years (mean age was 30.7 ± 15.1 years) with UCAR for at least 5 years were included. Most patients were females (66.9%), aged 10 to 30 years old (54.4%), and students (44.4%). In most patients (62.1%), AR symptoms started in the first decade of life and 88.5% of the patients had their diagnosis confirmed during this same period. Rhinosinusitis and asthma were in equal frequency the most common comorbidities. Half of the patients estimated self‐reported good general health (Table 1).

**Table 1:** Sociodemographic and clinical characteristics of UCAR patients

Variable	*N* (%)
Sex	
Male	103 (33.1)
female	208 (66.9)
Age, y	
10‐20	97 (31.2)
21‐30	72 (23.2)
31‐40	53 (17.0)
41‐50	51 (16.4)
51‐60	25 (8.0)
>60	13 (4.2)
Profession	119 (38.3)
Formal	18 (5.8)
Informal	138 (44.4)
Student	34 (10.9)
Unemployed/stay‐at‐home mothers	119 (38.3)
Age at first symptoms, y	
≤10	193 (62.1)
>10	118 (37.9)
Age at diagnosis confirmation, y	
≤10	275 (88.5)
>10	36 (11.6)
Comorbidities	
Rhinosinusitis	58 (18.6)
Asthma	56 (18.0)
Allergic conjunctivitis	4 (1.2)
Eczema	4 (1.2)
Patient self‐assessment of health	
Excellent	54 (17.4)
Good	162 (52.1)
Moderate	58 (18.6)
Bad	37 (11.9)
Frequency of symptoms	
Nasal congestion	234 (75.2)
Sneezing	233 (74.9)
Runny nose	212 (68.2)
Nasal itching	177 (56.9)
Posterior rhinorrhea	166 (53.4)
Ocular itching	152 (48.9)
Watery eyes	91 (29.3)

Abbreviation: UCAR, uncontrolled allergic rhinitis.

As listed in Table 2, multiple factors triggered the onset of symptoms in patients with UCAR, mostly including exposure to allergens, change in temperature, strong smell such as perfume (84.6%), humidity, and cigarette smoke (59.8%). The results of the SPT indicated that most patients were allergic to mites (82.0%), followed by cat (27.3%) and dog (26.7%) dander as well as mold (26.7%), pollen (20.3%), and cockroach (18.3%). Polysensitivity to allergens was by large more common (95.2%). Of all patients, 75.5% had persistent UCAR whereas the remainder had the intermittent type, and 59% reported worsening of the symptoms when working (not in Table 2). While 64% of the patients were compliant with their medication, the treatment was inadequate in 54% of them.

**Table 2:** Symptom triggers and skin allergy test findings

Variable	*N* (%)
Triggers of UCAR symptoms	
Exposure to allergens	310 (99.7)
Change in temperature	276 (88.7)
Strong smell/perfume	263 (84.6)
Humidity	224 (72.0)
Cigarette smoke	186 (59.8)
Physical exercise	53 (17.0)
Emotion/stress	66 (21.2)
Reaction to common allergens (SAT)	
Mite	255 (82.0)
Cat danger	85 (27.3)
Dog danger	83 (26.7)
Mold	83 (26.7)
Pollen	63 (20.3)
Cockroach	57 (18.3)
Peanut	27 (8.7)
Egg	17 (5.5)
Wheat	15 (4.8)
Soybean	11 (3.5)
Monosensitization	15 (4.8)
Polysensitization	296 (95.2)
Chicken	9 (2.9)
Parrot	3 (0.96)
Goat	2 (0.6)

Abbreviations: SAT, serum allergy testing; UCAR, uncontrolled allergic rhinitis.

Table 3 lists the treatments received and the reasons for failure. Nasal corticosteroid sprays (44.3%) and oral antihistamine medications (33.1%) were the most frequently prescribed types of medications. Most patients discontinued treatment without medical advice because they improved (30.2%) or felt no improvement at all (26%). Side effects including sleepiness and fatigue also prompted 14.1% and 4.2% of the patients, respectively, to discontinue treatment.

**Table 3:** Treatment received and reasons for discontinuation

Variable	*N* (%)
Types of treatment received	
Antihistamines	103 (33.1)
Nasal corticosteroid	138 (44.4)
Systemic corticosteroids	18 (5.8)
Nasal decongestants	92 (29.6)
Reaction to common allergens (SAT)	
Improvement	94 (30.2)
No improvement	81 (26.0)
Per physician recommendation	73 (23.5)
Financial reasons	4 (1.3)
Side effects	63 (20.3)
Sleepiness	44 (14.1)
Fatigue	13 (4.2)
Diarrhea	3 (1.0)
Lower limbs edema	3 (1.0)

Abbreviations: SAT, serum allergy testing.

The severity mean score of the UCAR naso‐ocular symptoms on the VAS is presented in Table 4. Nasal congestion (mean score: 7.0 ± 1.9), sneezing (6.9 ± 2.0), rhinorrhea (6.4 ± 1.9), and nasal itching (6.2 ± 1.9) were the most severe symptoms in our series.

**Table 4:** Severity of naso‐ocular symptoms on the visual analog scale in patients with UCAR

Symptoms	*N* (%)	Mean score
Nasal congestion	234 (75.2)	7.0 ± 1.9
Sneezing	233 (74.9)	6.9 ± 2.0
Rhinorrhea	212 (68.2)	6.4 ± 1.9
Itchy nose	177 (56.9)	6.2 ± 1.9
Posterior rhinorrhea	166 (53.4)	5.9 ± 1.9
Headache	161 (51.8)	5.9 ± 2.1
Itchy eyes	152 (48.8)	5.8 ± 2.0
Anosmia	79 (25.4)	5.4 ± 1.9
Watery eyes	91 (29.2)	4.8 ± 2.0
Shortness of breath	46 (14.8)	4.6 ± 2.5
Cough	58 (18.6)	4.5 ± 2.3
Wheezing	23 (7.4)	4.5 ± 2.2
Chest pressure	22 (7.1)	4.1 ± 1.8

## 4 DISCUSSION

The present study was designed to describe the clinical and allergen sensitization patterns in Congolese patients with UCAR and to determine symptom triggers. Nasal congestion, sneezing, and rhinorrhea were the most common clinical manifestations. Mites and pet dander were the leading sensitizing agents. Exposure to allergens was the main factor triggering the occurrence of symptoms. The majority of the patients were treated with nasal corticosteroid sprays often in association with oral antihistamine drugs.

### 4.1 UCAR and sex

Our finding that UCAR was more prevalent in females than males is in line with results of a previous population‐based study in this same setting^8^ and studies in Italy,^18^ Morocco,^19^ and Serbia.^20^ This finding likely suggests that cyclical hormonal variation in females increases nasal reactivity, as previously proposed by others.^21^ Nasal reactivity to histamine increases with estrogen blood level.^22^

### 4.2 UCAR and age

We found that most patients were 20 years old or younger. Past studies on AR in Africa^8^, ^23^ and elsewhere^15^, ^18^ reported similar findings. However, it is worth noting that selection bias by including a significant number of school children in the study population may have influenced our observation. The three medical institutions where the study was carried out may have been targeted by parents because they each have an attending ENT specialist. Another plausible explanation is the observation that allergic conditions likely start at an early age in developing countries, as a result of frequent and early use of antibiotics in children for various infections.^24^

In the present study, the first symptoms and the diagnosis of UCAR in most patients occurred at or below 10 years of age. The reason for this coincidence is not clear. We believe this may simply be due to the fact that around 10 years is when a child would be able to assess the severity of AR symptoms on the VAS.

### 4.3 UCAR and comorbidities

Sinusitis and asthma were the most commonly reported comorbidities. In Italy,^18^ conjunctivitis (53.7%) was the leading comorbidity, followed by asthma (37.8%) and sinusitis (13.7%). The absence of conjunctivitis in our patients likely resulted from the fact that they were not examined ophthalmologically.

### 4.4 UCAR symptom triggers

Exposure to allergens triggered UCAR in almost all of our patients. Other more common triggers included change in temperature, strong smell, and humidity. A similar trend was reported previously following a study conducted in the DRC by the United Nations Fund for Development.^25^ In that study, it was estimated that approximately 80% of illnesses in the general population was related to a bad environment. The same study reported that environmental risk factors may be the cause of health problems in approximately 30% of poor populations in sub‐Saharan Africa. Significant variations in temperature is known to result in nasal mucosa hyperreactivity. Indeed, nasal mucosa in patients with AR is particularly sensitive to temperature variations, which explains why exposure to repeated change in temperature increases the number, frequency, and severity of the symptoms.^26^ One particular environmental factor for the DRC and other sub‐Saharan countries the use of wood as the main source of household energy even in urban areas, with serious negative impact on air quality and health.^27^ Overall, environmental factors play an important role in the severity of nasal symptoms by inducing nasal hyperreactivity in patients with UCAR. They likely promote both IgE synthesis and allergy‐induced inflammation.^15^

Most patients were sensitive to mites, followed in much less proportions by cat and dog dander. Of note, mites are found worldwide, but are more abundant in countries with hot than mild climate.^8^, ^28^ Nyembue et al,^8^ in the same area but using a population‐based design, found mites and cockroach as the most common sensitizing allergens. Other studies in Africa have also reported mites, followed by pet dander and cockroach to be the most frequent allergens.^7^, ^26^, ^29^, ^30^ In contrast, pollen was the leading allergen, followed by mites in Italy.^18^

It is concerning that a substantial proportion our patients experienced symptom worsening when working, because it may diminish work performance.^31^, ^32^ Worsening of symptoms at work may be triggered by several factors including physical effort and irritating environmental conditions such as smoke, dust, and exposure to animals. It is important to note that chalk is still widely used in primary and secondary schools in the DRC and across sub‐Saharan Africa. It is a daily source of dust, but a neglected cause of symptoms worsening in schools.

### 4.5 Treatment side effects

The most frequent treatment side effects observed were sleepiness and fatigue, which resulted in treatment discontinuation in one out of five patients. A previous study by Keith et al^26^ also reported a similar finding. However, the authors estimated that such a behavior would result in poor quality of life.

### 4.6 UCAR and adherence to treatment

Although compliance to treatment was observed in 64% of our patients, treatment was inadequate in 54% of them due to side effects. The high rate of non‐adherence to treatment in our series likely resulted from the use of first generation antihistamines to control AR. Because these drugs have a low selectivity for H_1_‐receptors and are able to cross the blood‐brain barrier, a substantial number of patients will experience sleepiness, tiredness, diarrhea, and other symptoms as noted in this series.

In summary, this study highlights some aspects of UCAR in Congolese patients and provides information that may be used to develop optimal management programs for AR. Additional studies on a larger scale and in different areas of the country will be needed to provide a more comprehensive picture of the disease in DRC.

## CONFLICT OF INTERESTS

The authors declare that there are no conflict of interests.

## Data Availability

The data that support the findings of this study are available on request from the corresponding author. The data are not publicly available due to privacy or ethical restrictions.
